# The Incidence and Predisposing Factors of John Cunningham Virus-Induced Progressive Multifocal Leukoencephalopathy in Southern Finland: A Population-Based Study

**DOI:** 10.1093/ofid/ofz024

**Published:** 2019-02-22

**Authors:** Marge Kartau, Auli Verkkoniemi-Ahola, Anders Paetau, Maarit Palomäki, Rita Janes, Matti Ristola, Maija Lappalainen, Veli-Jukka Anttila

**Affiliations:** 1 Clinical Neurosciences, Neurology, Helsinki University Hospital and Helsinki University, Finland; 2 Department of Pathology (Neuropathology), Helsinki University Hospital Laboratory (HUSLAB), Helsinki University Hospital and Helsinki University, Finland; 3 Neuroradiology, HUS Medical Imaging Center, Helsinki University Hospital and Helsinki University, Finland; 4 Department of Oncology, Helsinki University Hospital and Helsinki University, Finland; 5 Department of Infectious Diseases, Inflammation Center, Helsinki University Hospital and Helsinki University, Finland; 6 Laboratory Services (HUSLAB), Department of Virology and Immunology, Helsinki University Hospital and Helsinki University, Finland

**Keywords:** HIV, JC virus, monoclonal antibodies, progressive multifocal leukoencephalopathy, rituximab

## Abstract

**Background:**

The aim of this study was to assess the prevalence, incidence rate (IR), predisposing factors, survival rate, and diagnostic delay of progressive multifocal leukoencephalopathy (PML) across medical specialties. Another objective was to survey how PML diagnosis was made in the studied cases.

**Methods:**

This is a cross-sectional retrospective observational study of PML cases across different medical specialties during 2004–2016 in the Finnish Capital Region and Southern Finland. Data were obtained from clinical records, clinical microbiology, pathology and radiology department records, and human immunodeficiency virus (HIV) quality register medical records.

**Results:**

A total of 31 patients were diagnosed with PML. The prevalence of PML was 1.56 per 100 000 people and the IR was 0.12 per 100 000 individuals per year during 2004–2016. Hematologic malignancies (n = 19) and HIV/acquired immune deficiency syndrome (n = 5) were the most common underlying diseases, and all patients who had malignant diseases had received cancer treatment. Before PML diagnosis, 21 (67.7%) patients were treated with chemotherapy, 14 (45.2%) patients with rituximab, and 1 patient (3.2%) with natalizumab. Two patients (6.5%) had no obvious immunocompromising disease or treatment. Neither gender, age, first symptoms, previous medication, nor underlying disease influenced the survival of PML patients significantly. The 5-year survival rate was poor, at less than 10%.

**Conclusions:**

The majority of PML patients in our study had a predisposing disease or had immunosuppressive or monoclonal antibody therapy. In the future, broader use of immunosuppressive and immunomodulatory medications may increase incidence of PML among patients with diseases unassociated with PML. Safety screening protocols for John Cunningham virus and PML are important to prevent new PML cases.

## Teaser

This is an observational study of PML cases during 2004-2016 where each case was identified and verified by clinical data by several specialties and biomarkers. Better safety protocols are recommended for all PML related medications.

Progressive multifocal leukoencephalopathy (PML) is a demyelinating disease of the central nervous system (CNS) caused by John Cunningham (JC) polyomavirus. John Cunningham virus (JCV) is a common and normally harmless human virus that establishes a life-long persistent infection in the renal tissue [1]. Progressive multifocal leukoencephalopathy occurs in immunocompromised individuals, as in acquired immune deficiency syndrome (AIDS) patients, and is seen in patients treated with immunomodulatory medications for chronic inflammatory and autoimmune disorders, hematological malignancies, or organ transplant recipients [2]. It is a rare side effect of monoclonal antibodies (mAbs) (such as natalizumab and rituximab) and other immunosuppressive medicines. The prevalence of PML in the general population was estimated at 0.22 per 100 000 individuals [3]. In a recent Swedish study, the incidence was 0.11 per 100 000 individuals per year [4]. In patients with autoimmune diseases rates of PML per 100 000 patients for systemic lupus erythematosus (SLE), rheumatoid arthritis (RA), and other connective tissue diseases, the incidence was 4.0, 0.4, and 2.0, respectively, compared with a rate of PML in the background population of 0.2 per 100 000 discharges. Rituximab therapy was associated with an increased prevalence of PML in RA patients (4 per 100 000). In patients with lupus, the risk of PML was higher than in RA [5].

Before the AIDS epidemic in the 1980s, PML was a rare disease. Interest in PML increased among neurologists in 2005 when its association with natalizumab was discovered [6]. Natalizumab is a mAb and is used in the treatment of highly active relapsing-remitting multiple sclerosis (MS). Other monoclonal therapies and drugs have also been reported to be correlated with an increased risk of PML [7]. Currently, MS patients who receive immunomodulatory therapies have become the third largest population of PML, after patients with human immunodeficiency virus (HIV) and hematological malignancies [8]. Respectively, the incidence of PML in HIV-infected patients has declined in the combined antiretroviral therapy era [9].

Among the HIV-infected patients, the known risk factor for development of PML is when CD4^+^ lymphocyte cell counts are less than 200 cells/µL [10]. Risk factors for HIV-negative persons have not been reported except for MS patients who receive immunomodulatory therapies. Natalizumab-treated MS patient PML risk has increased by presence of 3 risk factors: presence of anti-JC virus-antibodies in serum, treatment duration over 24 months, and prior treatment with immunosuppressant [11]. No particular duration or type of prior immunosuppressant use has been identified as a risk factor. Anti-JCV antibody levels in serum or plasma, measured as an index, further helps asses PML risk in JCV antibody-positive MS patients who receive natalizumab treatment [12]. On the other hand, JCV antibody-negative MS patients are unlikely to develop PML and can be treated with natalizumab for years if a safety protocol is followed.

We conducted a descriptive retrospective observational study of all incident cases of PML across different medical specialties during 2004–2016 in Helsinki University Central Hospital (HUCH) region, which covers the Finnish Capital Region and Southern Finland. The purpose of this study was to investigate the prevalence, incidence rate (IR), predisposing factors, and survival of PML. Another objective was to identify the correlates of the symptom-to-diagnosis interval (SDI) (diagnostic delay). The third objective was to survey how the PML diagnosis was made in the studied cases.

## METHODS

### Study Participants and Measures

The data were obtained from HUCH clinical records, clinical virology department records, pathology department records, radiology department records, and the HIV quality register. Patients with a suspected diagnosis of PML were examined by a radiologist, a neurologist, and an infectious disease physician before confirmation of a PML diagnosis.

Progressive multifocal leukoencephalopathy diagnoses were based on the American Academy of Neurology (AAN) recommendation [13]. Diagnoses of PML in the database were classified into 3 categories: (1) possible by typical neuroradiology and clinical findings without virological confirmation of JCV; (2) laboratory confirmed when patient had typical neuroradiology and clinical findings and JCV deoxyribonucleic acid (DNA) was detected in cerebrospinal fluid (CSF) by polymerase chain reaction (PCR); (3) definitive when patient had typical neuroradiology and clinical findings confirmed by histologic examination of a brain biopsy or autopsy specimen.

The JCV laboratory tests were mostly done in the Helsinki University Hospital Laboratory (HUSLAB), with few exceptions: 6 tests were done in the Karolinska University Laboratory and 5 tests were performed in the University of Turku Laboratory, which is now part of the TYKS Microbiology and Genetics Department. In the HUSLAB, quantitative JCV DNA PCR from plasma and CSF was performed as previously published [14].

In addition to neurological changes, suspicion of PML has to be supported by characteristic magnetic resonance imaging (MRI) features. All study patients had a 1.5T MRI scan performed. On T1-weighted images PML lesions are hypointense, and on T2-weighted and fluid-attenuated inversion recovery images hyperintense compared with normal white matter [15]. A rim of high signal on diffusion-weighted imaging (DWI) is characteristic. Typically, there is no mass effect. Magnetic resonance image findings that are highly indicative of PML have multifocal, asymmetric periventricular, and subcortical lesions with a predilection for the parieto-occipital regions. The subcortical U-fibers are commonly involved. Corpus callosum may also be involved [16]. Usually there is no gadolinium enhancement on MRI, but 15% of patients with HIV-associated PML and 40% of natalizumab-associated PML may exhibit gadolinium enhancement at the time of diagnosis [13].

The SDI was calculated as the time between onset of symptoms and establishment of diagnosis usually by examination of JCV DNA from CSF samples or brain biopsy samples. The prevalence and IR were calculated per 100 000 person-years during 2004–2016. The IR of PML was calculated using the number of enrolled patients and the duration of follow-up (13 years). Statistical analysis was performed using SPSS versions 23 and 24. Due to uneven distribution of study data, statistical analysis was performed with non-parametric tests. Descriptive analysis included median value and range. Differences between 2 groups were assessed with Mann-Whitney *U* test. Difference between more than 2 groups was assessed with Kruskal-Wallis test. Survival duration was determined using Kaplan-Meier approach. The 5-year survival rate was calculated from the point of diagnosis to the end of year 2017. The study protocol was approved by research board of the Inflammation Center at the Helsinki University Hospital on March 29, 2017.

## RESULTS

### Descriptive Statistics

This study had a total of 31 patients (N = 31): 17 males (54.8%) and 14 females (45.2%). The median age at the time of PML diagnosis was 62 (minimum age was 22 and maximum 83 years).

### Progressive Multifocal Leukoencephalopathy Diagnoses

In total, 9 patients (29%) had definitive diagnoses of PML, 19 patients (61.3%) had laboratory-confirmed diagnoses, and 3 (9.7%) patients had possible diagnoses (Table 1). Two of the patients with possible diagnoses had a negative CSF JCV PCR result, and from 1 patient CSF sample was not obtained. Two of the possible diagnoses patients had hematological malignancies and 1 had HIV. All 3 had typical clinical pictures and radiological findings of PML. John Cunningham virus DNA was positive in 23 patients’ CSF (74.2%) and negative in 4 patients’ CSF (12.9%). A CSF sample was not taken in 4 cases (12.9%).

**Table 1.** Characteristics of PML Patients

Characteristics	Total	Definite PML	Probable PML	Possible PML
Patients, n	31	9	19	3
Sex, M/F	17/14	3/5	12/8	2/1
Underlying disease				
Hematologic malignancies^a^		5	12	2
HIV/AIDS		1	3	1
Rheumatologic diseases		1	2	0
Neurologic diseases		0	2	0
Previously healthy		1	1	0
Brain biopsy	4	4	0	0
Neuropathologically confirmed PML	8	8	0	0
Brain MRI	31	9	19	3
Monoclonal antibody treatment^b^	15	2	12	1
Immunosuppressive treatment^c^	25	7	16	2
SDI, mean, days	86, 42	104	86	43
Survival at the end of 2017	5	0	5	0

Abbreviations: AIDS, acquired immune deficiency syndrome; HIV, human immunodeficiency virus; MRI, magnetic resonance imaging; PML, progressive multifocal leukoencephalopathy; SDI, symptom-to-diagnosis interval.

^a^Eight patients with B-cell lymphomas, 4 patients with chronic lymphatic leukemia, 2 patients with Waldenstrom’s macroglobulinemia, 2 myeloma patients, 1 patient with polycythemia vera, 1 with acute myeloid leukemia, and 1 patient with mastocytosis.

^b^Rituximab, natalizumab.

^c^Twenty-one patients had received cancer chemotherapy. Twenty-one patients were treated with cortisone. Four patients were treated with other immunosuppressants such as methotrexate, azathioprine, mycophenolic acid, hydroxychloroquine, and cyclosporine.

### Neuroimaging in Progressive Multifocal Leukoencephalopathy

In our study, there were 21 patients (67.7%) with no gadolinium enhancement lesions and 8 patients (25.8%) with enhancing lesions. Gadolinium enhancement lesion data were omitted from 2 patients. Six patients (19%) had presentation of PML in posterior fossa, 2 of them had only cerebellar lesions, and 4 had both supra- and infratentorial lesions.

### Procedures

Autopsies were performed on 10 patients, and complete neuropathological examinations and confirmations for PML diagnoses were done on 9 patients (29%). Brain biopsies were performed on 4 patients (12.9%) to obtain PML diagnoses.

### Prevalence and Incidence Rate of Progressive Multifocal Leukoencephalopathy

**Figure 1.**
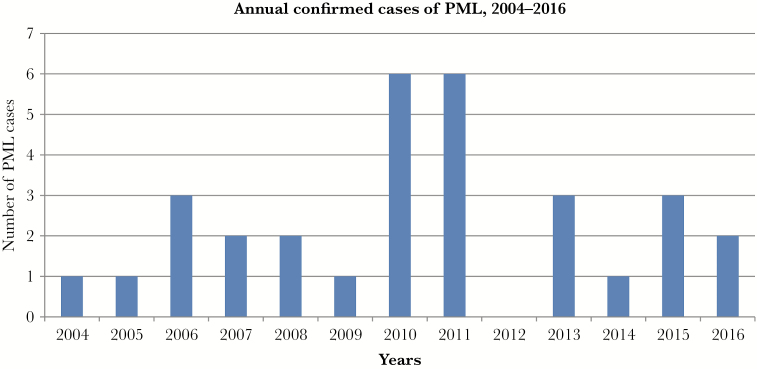
Annual cases of progressive multifocal leukoencephalopathy (PML).

There were 30 patients from the Finnish Capital Region and the HUCH district during 12 years (Figure 1). One patient was consulted from outside the region. The population in this area is 1 919 254 (December 31, 2015) [17]. The total number of individuals with the disease divided by 100 000 persons in population is 1.56 in the HUCH area during the 13 years of study. The IR per year is 0.12 per 100 000 person-years during 2004–2016.

### Underlying Diseases

Most of the PML patients had been immunosuppressed due to underlying diseases or immunomodulatory therapies. In this study, there were 19 patients with hematologic malignancies (61.3%): 8 patients with B-cell lymphomas, 4 patients with chronic lymphatic leukemia (CLL), 2 patients with Waldenstrom’s macroglobulinemia, 2 myeloma patients, 1 patient with polycythemia vera, 1 with acute myeloid leukemia, and 1 patient with mastocytosis. Most of hematologic malignancies (84.2%) were lymphoid neoplasms. Five patients had HIV/AIDS (16.1%), 4 of them with CD4 counts below 200 cells per mm^3^ at PML diagnosis and 1 of them after developing immune reconstitution syndrome 5 months after starting antiretroviral therapy at CD4 count 384 cells per mm^3^. Three patients had rheumatologic disease (9.7%): 2 SLE and 1 dermatomyositis. Two patients (6.5%) had underlying neurological disease: MS and primary CNS vasculitis. Two patients (6.5%) had no obvious immunocompromising disease or treatment (Table 2). One of these patients had diabetes mellitus type II and suffered from alcoholism.

**Table 2.** Underlying Disease

Characteristics	Frequency	Percent
Hematologic malignancies		
Lymphoid neoplasms	16	51.6
Myeloid neoplasms	3	9.6
HIV/AIDS	5	16.1
Rheumatologic disease	3	9.6
Neurologic diseases	2	6.5
Others	2	6.5
Total	31	100

Abbreviations: AIDS, acquired immune deficiency syndrome; HIV, human immunodeficiency virus.

### Immunosuppressive Treatment

Twenty-one (67.7%) patients had received cytostatic treatment before a PML diagnosis, 21 patients (67.7%) were treated with cortisone, and 4 (12.9%) patients were treated with other immunosuppressants such as methotrexate, azathioprine, mycophenolic acid, hydroxychloroquine, and cyclosporine. Fifteen patients had mAb therapy before PML diagnosis: 1 MS patient (3.2%) received natalizumab and 14 patients (45.2%) received rituximab (Table 1). Seven patients (50%) who received rituximab treatment had B-cell lymphoma, 4 (28.6%) had other hematologic malignancies, and 3 (21.4%) had rheumatologic disease as an underlying disease. Lymphoma patients and hematologic patients had rituximab infusions as part of their chemotherapy regimen. Three patients (21.4%) had their first PML symptoms before the end of rituximab treatment. The mean times from last rituximab infusion to PML diagnosis and from last infusion to first PML symptoms were 5.86 and 3.07 months, respectively. The mean time from first rituximab infusion to PML symptoms was 27.2 months, minimum time was 1 month, and maximum time was 102 months.

### Clinical Course and First Symptoms of Progressive Multifocal Leukoencephalopathy

In our study, the first symptoms of PML were shown as cognitive and behavioral changes for 13 patients (41.9%), visual disturbances for 11 patients (35.5%), language problems for 8 patients (25.8%), motor weakness for 6 patients (19.4%), ataxia for 6 patients (19.4%), headache for 2 patients (6.5%), and 1 patient (3.2%) had epileptic seizures, nausea, and vertigo. Some patients had more than 1 first symptom.

### Symptom Diagnosis Interval

The SDI mean was 86.42 days and median was 57 days. The minimum and maximum times were 17 and 450 days, respectively, and range was 433 days.

### Survival

Over the course of this study, 5 patients (16.1%) were alive at the end of year 2017 and 26 patients (83.9%) were deceased. The surviving patients were as follows: 1 with MS diagnosis, 1 with SLE, 2 patients with AIDS, and 1 patient with Waldenstrom’s macroglobulinemia. Three patients have survived over 5 years, making the 5-year survival rate 9.7%. The minimum time from first PML symptoms to death was 49 days, and the maximum time was 1784 days, the mean was 233.9, and the median was 130 days. Nine patients (29%) survived 1 year, and 22 patients (71%) died during the first year after a diagnosis of PML was made. No statistically significant differences in survival of PML were observed between women and men or between age groups. The time from first symptoms to PML diagnoses, underlying disease, or previous medication did not influence patient survival.

## DISCUSSION

Our study showed that the prevalence of PML was 1.56 in The Finnish Capital Region and Southern Finland during 13 years, and the IR per year was 0.12 per 100 000 person-years during 2004–2016. The results are consistent with the previous reports from the multinational population of rheumatological patients with PML and the national incidence of PML in Sweden [4, 5].

To develop PML, a patient has to have a predisposing disease, such as suppressed cellular immunity, for example HIV infection or B-cell disease, in addition to the JC virus. Progressive multifocal leukoencephalopathy may also be an adverse effect of immunosuppressive therapy or therapeutic mAb, such as natalizumab and rituximab regimens. In our study, half of the patients had mAb therapy and 67.7% had chemotherapy. Seventy-seven percent of patients had classic predisposing diseases such as hematologic malignancies or HIV/AIDS, and all the patients who had malignant diseases had received immunomodulatory treatment before PML. Only 2 patients (6.5%) had no obvious immunocompromising disease or treatment.

Progressive multifocal leukoencephalopathy is not easy to diagnose because at the beginning, the disease may be asymptomatic [18] and early symptoms are difficult to recognize and detect, because they can be cognitive and behavioral. The symptoms result from brain lesions in corresponding areas, such as frontal lobes for motor weakness, occipital lobes for visual field deficits, and cerebellum for ataxia [19]. Clinical features can vary, but hemiparesis, ataxia, gait disturbance, visual deficits, and cognitive changes are prevalent with limb weakness being the most common with HIV and MS-associated PML [15, 19]. Cognitive and gait disturbances and visual deficits are the next most common. In our study, the most common first symptoms of PML were cognitive and behavioral changes, which were observed in 42% of cases. The next most common were visual disturbances and language problems. Motor weakness was only observed in 19% of cases. Some patients had more than 1 first symptom. Progressive multifocal leukoencephalopathy first symptoms did not influence the outcome or survival of patients covered by our study.

In addition to the neurological symptoms, diagnosis of PML has to be supported by characteristic imaging features. Brain MRI is a valuable tool for confirming a clinical suspicion of PML in addition to laboratory diagnostics. The MRI appearance of PML is single or multifocal white matter lesions with involvement of subcortical U-fibers. Progressive multifocal leukoencephalopathy lesions typically involve white matter, but gray matter involvement has also been observed [20]. Lesions become large and confluent as the disease progresses. Usually there is no mass effect or gadolinium enhancement [16]. However, gadolinium enhancement has been observed in HIV- and natalizumab-associated PML lesions [15], and it was observed in 26% of our cases. The PML may occur anywhere in the brain, but the frontal and parieto-occipital regions are most commonly affected [15].

According to the AAN recommendations, it is important to associate the clinical and imaging abnormalities with the evidence of JCV in the brain to make a PML diagnosis [13]. Although JCV antibody testing in plasma can be used to assess PML risk, it has currently no diagnostic value after the onset of PML. Instead, a valuable diagnostic tool is a sensitive laboratory test to detect intrathecal JCV DNA in CSF by highly sensitive PCR. Cerebrospinal fluid examination also helps to exclude other diagnoses. Despite the high sensitivity of PCR, a negative test result does not rule out PML [13]. In HIV-infected patients, the viral load in CSF is high and sufficient for diagnosis. Acquired immune deficiency syndrome treatment decreases viral load and makes laboratory-confirmed diagnosis difficult. In addition, in patients with MS and other autoimmune diseases, the viral loads are low [13]. This creates a need to take multiple CSF samples despite a clinically strong suspicion of PML because initial samples are falsely negative. In our study, JCV DNA was positive in 23 patients’ CSF (74.2%) and negative in 4 patients’ CSF (12.9%). Cerebrospinal fluid samples were taken once from 16 patients (51.6%), twice from 9 patients (29%), thrice from 2 patients (6.5%), and not taken at all in 4 cases (12.9%).

A brain biopsy is indicated in patients where the CSF cannot be obtained or is negative and the MRI is not characteristic of the disease. Infected brain cells can be demonstrated by immunohistochemistry, in situ hybridization, or PCR analysis [21].

An early PML diagnosis is important especially in natalizumab-treated MS patients, because prompt restoration of the immune system is the only intervention that can improve survival chances [22]. The median SDI in literature has been reported as 74 days (range, 1–1643) [23]. The mean SDI time in our study was 86 days and the median time was 57 days (range, 17–450). Two patients with a hematological disease had the shortest SDI values (17–21 days). Two patients with a rheumatological disease (SLE and dermatomyositis with SDI 450 and 168 days, respectively) and 1 with hematological disease (SDI 177 days) had the longest SDI values.

Progressive multifocal leukoencephalopathy is a highly debilitating disease with a high mortality rate. For most patients with existing serious immunosuppressive diseases, the detected PML diagnosis leads to death. Within the first 3 months of diagnosis, the mortality rate is 30%–50% [24]. For patients with HIV-associated disease, the 2-year mortality rate is 50%–60%, despite highly active antiretroviral therapy and adjunctive treatments [25, 26]. Survival of PML patients with hematologic malignancies is very poor, with approximately 90% mortality within 2 months of diagnosis [27]. In PML associated with iatrogenic immunosuppression, removal of a causative agent together with supportive care has improved survival rate up to 77% [22]. Reversal of natalizumab immunosuppression by plasmapheresis to restore immune function has improved the outcome of MS-associated PML [15]. Predictors for favorable outcomes in natalizumab-associated PML cases were younger age, lower Expanded Disability Status Scale (EDSS) score prior to diagnosis, shorter diagnostic delay, and more localized brain lesion on MRI at the time of diagnosis [28]. Our results showed that patient gender, age, or underlying disease did not significantly influence the outcome or survival of patients with PML. We had only 1 natalizumab-associated PML case.

This study was limited by being single-centered and using an observational retrospective method. On the other hand, most of the PML diagnoses were made in co-operation between a radiologist, a neurologist, a pathologist, and an infectious physician according to AAN recommendation: 29% had definite diagnoses of PML, and 61.3% had laboratory-confirmed PML diagnoses. Only 1 diagnosis was made post mortem.

In the future, broader use of immunosuppressive and immunomodulatory medications in the fields of rheumatology, neurology, gastroenterology, and dermatology may increase incidence of PML among patients with diseases unassociated with PML. With a growing number of available therapies, patients are multiexposed to different drugs in the course of their disease, making it difficult to evaluate individual patient’s total risk of PML and different medications risk of causing PML as a severe side effect. Each treatment-associated case of PML should be assessed and reported, regardless of the patient’s previous medical treatment. The risk-benefit ratio of mAb therapies should be carefully assessed, especially if these treatments are used off-label. Progressive multifocal leukoencephalopathy risk for MS patients is well known, and disease-modifying therapies have been classified by their PML risk [29]. For example, natalizumab has high PML risk. According to latest Tysabry Safety Update information, the global overall incidence of PML in natalizumab-treated patients is 4.15 per 1000 patients (95% CI = 3.87 to 4.44 per 1000 patients). Much lower risk exists for fingolimod and dimethyl fumarate. There are approximately 9000 MS patients in Finland, 2700 of which receive different MS disease-modulating drugs in HUCH. Over an 11-year period, there have been approximately 70–80 patients per year who receive 1 natalizumab intravenous infusion per month, during which only 1 PML case has been recorded in the Finnish capital region and Southern Finland. After that 1 case, we have considerably improved the natalizumab safety screening protocol. This has proven to be effective despite high costs. There have been no other PML cases among our MS patients. For other than MS patients’ treatments, there are no such classification and safety protocols. In our study, there were 14 patients who had rituximab therapy, 80% of which had B-cell lymphomas or hematologic malignancies as underlying diseases and 20% had rheumatologic diseases. All of these patients were not screened for PML risk. It may be argued that this is acceptable in cancer patients because rituximab therapy greatly improved likelihood of event-free survival, but for rheumatology patients this is questionable.

## CONCLUSIONS

All physicians who use mAb treatments should be aware of risks, diagnosis, and management of PML. Proper information sharing to both patients and medical staff plays an essential role in the prevention, early detection, and risk factor screening. If PML risk is acknowledged, preventative actions are required, such as switching therapies, regular brain MRI, and symptoms monitoring. If new symptoms occur, PML should be considered and appropriate diagnostic testing should be done as soon as possible.

## Note

***Potential conflicts of interest.* **All authors: No reported conflicts of interest. All authors have submitted the ICMJE Form for Disclosure of Potential Conflicts of Interest.
